# Seasonal changes in population structure of the ambrosia beetle *Xylosandrus compactus* and its associated fungi in a southern Mediterranean environment

**DOI:** 10.1371/journal.pone.0239011

**Published:** 2020-09-11

**Authors:** Antonio Gugliuzzo, Giulio Criscione, Antonio Biondi, Dalia Aiello, Alessandro Vitale, Giancarlo Polizzi, Giovanna Tropea Garzia

**Affiliations:** Department of Agriculture, Food and Environment (Di3A), University of Catania, Catania, Italy; Institut Sophia Agrobiotech, FRANCE

## Abstract

Exotic ambrosia beetles are increasing in Europe due to global trade and global warming. Among these xylomycetophagous insects, *Xylosandrus compactus* (Eichhoff) (Coleoptera: Curculionidae) is a serious threat for several Mediterranean host plants. Carob trees growing in Sicily (Italy) have been extensively attacked by beetles leading to rapid tree decline. Although *X*. *compactus* has been found in Europe for several years, most aspects of its ecology are still unknown. We thus studied the population structure and dynamics of *X*. *compactus*, together with its twig size preference during a sampling of infested carob trees in south east Sicily. In addition, fungi associated with insects or galleries were isolated and characterized. The results showed that, in this newly-colonized environment and host plant, adult *X*. *compactus* overwinters inside twigs and starts to fly and reproduce in mid spring, completing five generations before overwintering in late fall. The mean diameter of carob twigs infested by the beetle varied significantly over the seasons, with the insect tending to infest larger twigs as season progresses. The mean number of adults/gallery was 19.21, ranging from 6 to 28. The minimum temperature significantly affected the overwintering adult mortality. *Ambrosiella xylebori* and *Fusarium solani* were the main symbionts associated with the pest in this study. *Acremonium* sp. was instead recorded for the first time in Europe inside *X*. *compactus* galleries. Several other fungi species were also found for the first time in association with *X*. *compactus*. Our findings provide useful insights into the sustainable management of this noxious pest.

## Introduction

The number of exotic insect pests established in Europe is constantly increasing, mainly due to global trade and global warming [1–7]. These organisms often cause economic damage as well as an increase on pesticide applications with the consequent side effects toward non-target organisms, the environment and human health [8–12]. Among insect pests which have invaded the Mediterranean Basin over the last decades, alien wood-boring beetles are of primary importance due to the high diversity of host plants and the suitable climate [13–17]. The mild winters followed by dry and warm summers of Mediterranean regions, favour the spread and establishment of alien Scolytinae [18], including several xylomycetophagous ambrosia beetle species belonging to the genus *Xylosandrus* [19]. In particular, four of these species are native to Asia and are now widespread in Italy. *Xylosandrus morigerus* (Blandford) is known as a plant nursery pest, while *X*. *crassiusculus* (Motschulsky) and *X*. *germanus* (Blandford) are considered pests of various cultivated and wild host species [20–23].

Since its first report in 2012 [24, 25], *X*. *compactus* (Eichhoff), also known as the black twig borer, has been reported as an emerging pest for several plants of the Mediterranean maquis [26], as well as for various trees and ornamental shrubs that are widespread in southern Europe [19, 27, 28]. Among these, *Laurus nobilis* L. [25] and carob tree (*Ceratonia siliqua* L.) have been reported as preferred host plants [29]. Unusual heavy pest infestations on large branches and trunks, associated with serious decline and wilting, have been recently observed on carob trees in Sicily (Southern Italy) [29–31]. Carob is a thermophilous arboreal species characteristic of the olea-lentisc and carob groups (belonging to the *Oleo sylvestris-Ceratonion siliquae* alliance) [32] and it is a widespread and long-living tree in Mediterranean woodland vegetation [33, 34]. This tree species, largely diffused in dry areas of Sicily, provides to farmers several products and by-products including the flour extracted from the seeds (Locust Bean Gum, LBG) used in food industry as thickening agent (E410) [35–37].

*Xylosandrus compactus*, as well as the other ambrosia beetles, develop by feeding exclusively on fungi cultivated by females inside galleries [38–40]. In insect-fungus mutualisms, symbiotic fungi can degrade the defensive substances of a plant and/or directly produce antagonistic compounds against other microorganisms that may co-occur in the galleries [41]. Among the symbiotic fungi reported in association with *X*. *compactus*, three species seem to be the most recurrent, i.e., *Ambrosiella xylebori* Brader ex Arx, *A*. *macrospora* (Francke-Grosm.) L.R. Batra and *Fusarium solani* (Mart.) Snyd. & Hans [26, 42–45]. On the other hand, several other fungal species, including *Geosmithia pallida*, *Epicoccum nigrum* and *Bionectria* sp., have been found in association with the black twig borer on Mediterranean maquis plants [26]. However, the symbiotic community composition may be spatio-temporal dependent. Bateman et al. [46] isolated *A*. *xylebori* almost exclusively from the mycetangium (a fungus spore-carrying organ), and *Fusarium* spp. mainly from the body surface, clearly demonstrating that the different fungi are spatially segregated on the insect’s body. Skelton et al. [47] demonstrated that closely related symbionts are interchangeable by offering alternative fungal symbionts from different *Ambrosiella* clade in experimental galleries inoculated with *X*. *compactus*. However, Li et al. [48] showed that aposymbiotic specimens, deprived of *Ambrosiella*, in any case develop empty mycetangia.

Although *X*. *compactus* has been in Europe for several years, causing much damage to wild and cultivated plants, its population structure over the seasons in the newly-invaded areas has not yet been investigated. Similarly, there is little information on the fungal communities of *X*. *compactus* in such areas. The annual population trend of the beetle was thus monitored, and the fungi associated with the insect or occurring inside the infested galleries were identified and characterized.

## Materials and methods

### Beetle samplings and dissection of the samples

The study was carried out by sampling unmanaged carob trees (var. Latinissima) from the beginning of November 2017 to the end of December 2018, in the town of Scicli (18m a.s.l., 36°45’45.0"N, 14°38’40.3"E) located in south east Sicily (Italy). This sampling site was characterized by an area of about 10 ha of a semi-urban environment next to a natural landscape, typical of the Mediterranean environment, where many natural carob trees grow spontaneously without any anthropic intervention. In this location, the *X*. *compactus* first appeared in summer 2017 [29]. Climatic data were provided by the Sicilian Agrometeorological Service (SIAS). The data (minimum, maximum and daily average temperatures) were obtained from the nearest climatic station located 5 km from the sampling site (51m a.s.l., 36°45’41.3"N, 14°40’50.5"E).

A yearly population trend of *X*. *compactus* on carob trees was estimated by sampling and dissecting twigs (20 twigs/biweekly) that showed infestation signs, such as the presence of an ambrosia beetle entrance hole, wilting, defoliation and wood necrosis near the entrance holes [22, 29]. Samplings were carried out every 15 days in order to increase the likelihood of sampling all the different biological stages of each pest generation, given this beetle completes its life cycle in about one month [38]. For each sampling date, four twigs/tree (approximately 1.5–2 m above the ground) were sampled from all the cardinal points of five randomly chosen trees located at a distance of about 50m from each other. Each sampling included ten small twigs (diameter ≤7 mm; length ≤ 50 cm), usually infested by one or few females, and ten larger twigs (diameter ≥8 mm; length >50 cm), usually attacked by multiple females [22,30,38]. Twig diameter was measured before the samplings using a Vernier caliper.

Twigs were cut using secateur-type scissors, an extendable pruner or other cutting tools, and transferred to the laboratory and dissected the same day under environmental room conditions (25 ± 2 °C and 60 ± 10% R.H.). Specimens found inside each brood chamber were counted under a stereomicroscope, and the adults were identified by their main specific morphological traits [49]. For each infested twig, the diameter of the twig at the entrance holes and the number of biological stages (eggs, larvae, pupae, and adults) inside each gallery were recorded. Dead specimens inside the galleries were also counted. For galleries infested by adults only, the mean number of specimens/gallery was calculated.

### Fungal isolation and identification

Fungi that were inhabiting the beetle’s external body surface or growing in the infested twig galleries of each sampled twig were isolated. Sections of symptomatic twig tissues were excised from the lesions surrounding the beetle gallery and disinfected with 1.5% sodium hypochlorite solution, rinsed in sterile water, and placed on potato dextrose agar (3.9% PDA, Oxoid). To prevent bacteria growth, medium plates were amended with 100 mg/liter of streptomycin sulphate (Sigma-Aldrich). Samples were incubated at 25 ± 1°C, or until the fungal growth was evident. Fungi inside the galleries were also isolated by scraping off a portion of the fungal biomass with a sterile wood-stick and transferring it onto PDA. For isolation of the beetle fungal communities, 2–3 not disinfected beetles per sample were plated on PDA. Per each sampling date, from two to six fungal isolates were obtained from twigs or insects.

The fungi colonies were transferred onto new plates to obtain pure cultures. Single conidium or hyphal tip culture fungi were then established on PDA for all fungal colonies. After being stored in an incubator in the dark at 25 °C for up to two weeks, morphotypes were assigned based on macromorphology (i.e., color, size comparison/growth rate, texture). These cultures were separated into possible ambrosia and other fungi by examining the colony characteristics [44, 46]. Representative isolates of mean species were also identified with molecular analysis. All isolates were stored at -20 °C in 20% (v/v) glycerol at the Department of Agriculture, Food and Environment of the University of Catania, Italy.

Molecular identification of the fungal isolates was performed by sequencing internal transcribed spacer regions of the rDNA and 5.8S region (ITS). Genomic DNA was extracted from 35 isolates using the Wizard Genomic DNA Purification Kit (Promega Corporation, WI, USA). The ITS of the nuclear ribosomal RNA operon was amplified with primers ITS5 and ITS4 for all isolates and species [50].

The PCR products were sequenced in both directions by Macrogen Inc. (South Korea). The DNA sequences generated were analysed, and consensus sequences were computed with Mega 7 [51]. BLAST searches were used to compare the obtained sequences with other sequences in the NCBI database.

### Data analyses

The raw insect data obtained were preliminarily examined in order to detect the first two dates in a row with adults only infesting the sampled twigs, i.e., the beginning of the insect reproductive diapause. Mid-December 2017 (see the Results section) was thus set as the starting sampling date for analysing the yearly structure of the *X*. *compactus* population. Data were thus divided into the following four seasons: from Dec 15 2017 to March 14 2018 (winter), from March 15 2018 to Jun 14 2018 (spring), from Jun 15 2018 to Sep 14 2018 (summer) and from Sep 15 2018 to Dec 14 2018 (autumn).

Raw datasets were then tested for normality and homogeneity of variance using Kolmogorov-Smirnov D test and Cochran’s test, respectively, and no data transformation was needed. Data were analysed by factorial ANOVA (at a level of significance of p ≤ 0.05), using season as the independent factor. The dependent variables were the specimen numbers belonging to each biological stage (egg, larval, pupal and adult) in the whole sample, the mean diameter of infested twigs, and the number of adults/gallery. A Bonferroni *post-hoc* test was conducted in order to compare the mean diameter of infested twigs over the different seasons. The temperature dependent mortality of overwintering adults was described by a linear regression model, studying the proportion of dead adults (number of dead adults/number of sampled adults) per sampling date as a function of the minimum temperature trend of the two weeks before the sampling. The trend was estimated by calculating, per sampling date, the mean minimum daily temperature of the fourteen days preceding each sampling. Statistical analyses were carried out using SPSS 22.0 software (IBM Corp., Armonk, NY, USA).

## Results

### Yearly beetle population structure

**Fig 1:**
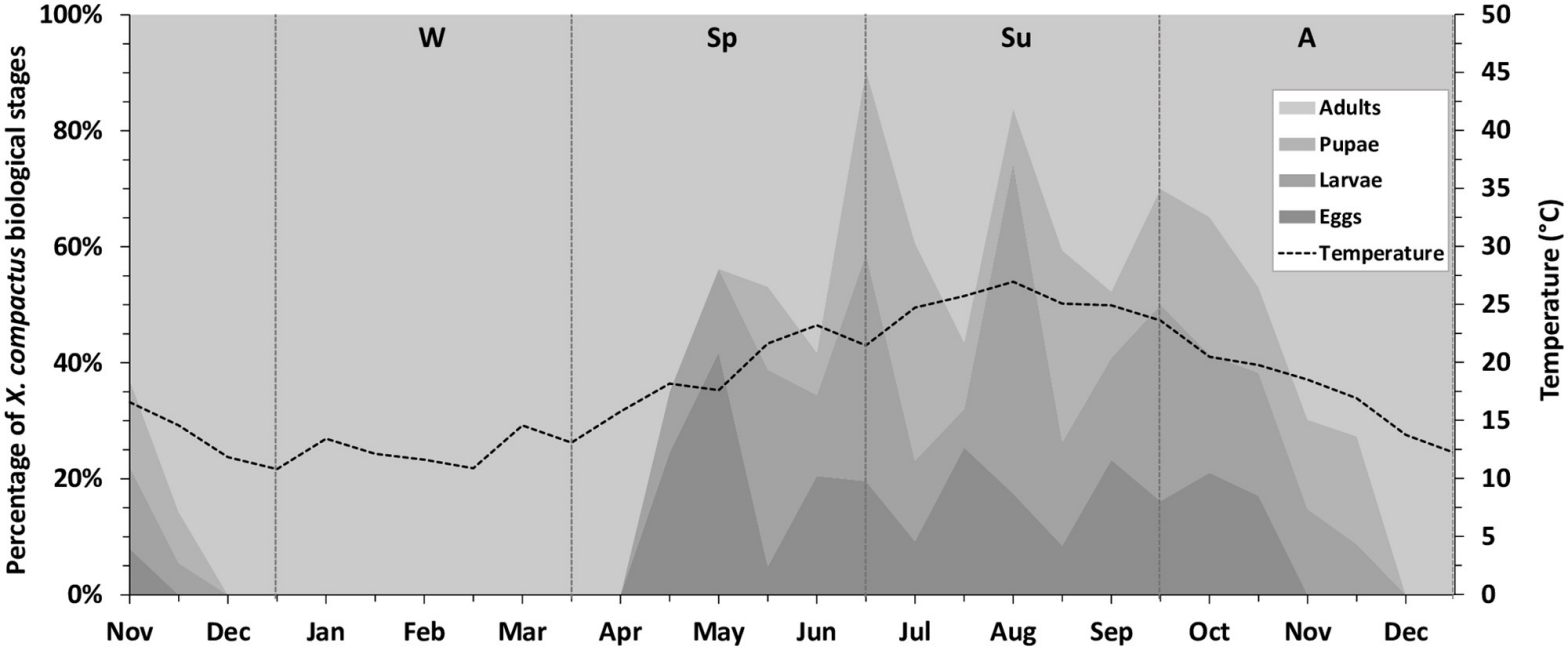
Annual trend of the *Xylosandrus compactus* population structure on carob trees in Sicily (Southern Italy) from November 2017 to December 2018. Mean percentage of eggs, larvae, pupae and adults of *X*. *compactus* found inside the sampled carob twigs. During winter only inactive (overwintering) adults were found. The dashed line represents the biweekly mean temperature trend. Seasons: W = winter, Sp = spring, Su = summer, A = autumn.

During the first three sampling dates, there was a decreasing trend in the proportion of immature beetles, i.e., 37, 14.3 and 0% of the sampled individuals were juveniles, in mid-November, late-November and early December 2017, respectively (Fig 1). In line with this, on Dec 15 2017, the sampled beetles were all adults, and therefore this was considered as the starting date for the yearly analyses. The population trend of *X*. *compactus* was affected by the season, as shown in Fig 1. The specimen percentage of the four developmental stages varied significantly over the seasons (eggs: *F*_*3*,*198*_ = 3.446, *p* = 0.018; larvae: *F*_*3*,*198*_ = 10.405, *p*< 0.001; pupae: *F*_*3*,*198*_ = 7.991, *p*< 0.001; adults: *F*_*3*,*198*_ = 3.094, *p* = 0.028).

During the winter (from mid December 2017 to early April 2018), only adults were found inside the galleries. Beetle eggs occurred from April to November 2018 with five major peaks: in early May (41.8%), mid-June (20.5%), late July (25.4%), early September (23.3%) and early October 2018 (21.1%). Larvae were sampled from late April to late November with four major peaks in mid-May (33.9%), late June (39.2%), early August (57%) and late September (33.8%), and a minor one in late October 2018 (21.2%). The amount of pupae, recovered from late May to late November, followed the same trend as the larvae (Fig 1). Adults were always found infesting the sampled twigs, however their numbers were constant during the winter but variable from spring to autumn, with major peaks in early June (58.2%), late July (56.5%), and early September 2018 (47.7%).

**Fig 2:**
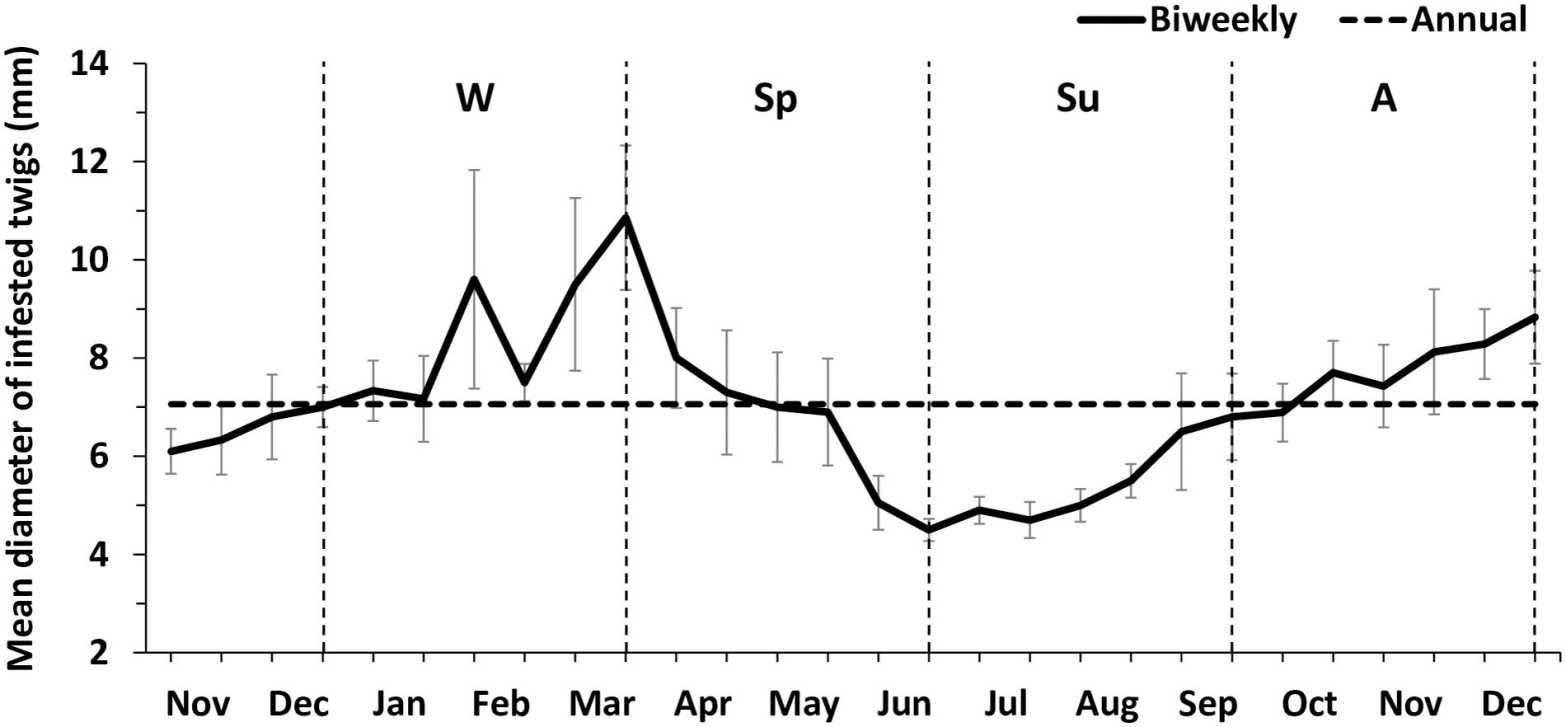
Mean (±SE) diameter of carob twigs infested by *Xylosandrus compactus* from November 2017 to December 2018 in Sicily (Southern Italy). Seasons: W = winter, Sp = spring, Su = summer, A = autumn.

The mean diameter of infested carob twigs was 7.14 mm (± 0.34), but varied significantly according to the season (*F*_*3*,*198*_ = 17.061; *p*< 0.001) (Fig 2). Results of the *post-hoc* Bonferroni test showed that the mean diameter of infested twigs during the summer was significantly different from the other seasons (summer vs spring, summer vs autumn, and summer vs winter: *p*< 0.001). However, no significant difference was found between the mean infested diameter during spring, autumn and winter (spring vs autumn: *p* = 0.984; spring vs winter and autumn vs winter: *p* = 1.000). Specifically, during the winter, it ranged from 7.17 ± 0.87 mm in January to 10.86 ± 1.47 mm in March. While, during the summer, the mean diameter of infested twigs ranged from 4.50± 0.22 mm in June to 5.50 ± 0.34 mm in August. This value also decreased during the spring and was higher during the winter (Fig 2).

There was no significant difference between the number of adults/gallery among the seasons (*F*_*3*,*136*_ = 0.137; *p* = 0.938). The mean value of adults/gallery during the monitoring period was 19.21± 0.40 (mean ±SE), ranging from 18.91± 0.80 in winter to 19.58 ± 0.70 in autumn. Specifically, the minimum and maximum number of adults of *X*. *compactus* found inside a single gallery was 6 and 28, respectively.

**Fig 3:**
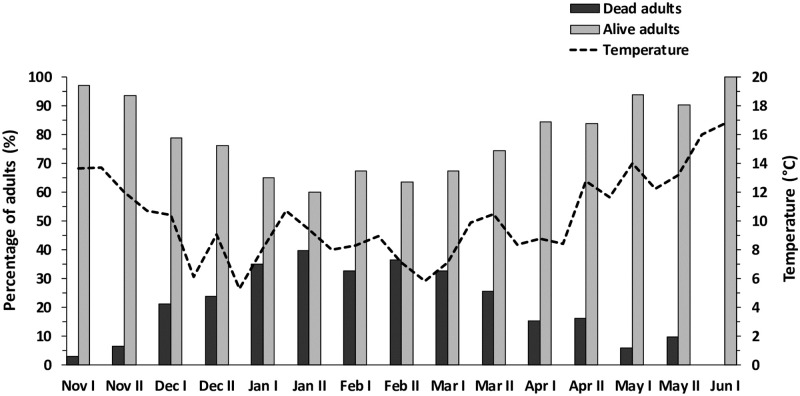
Mortality of *Xylosandrus compactus* overwintering adults inside carob twigs in Sicily (Southern Italy). Mean percentage of live and dead adults found infesting the sampled carob twigs. The dashed line represents the mean minimum temperatures of the period.

**Fig 4:**
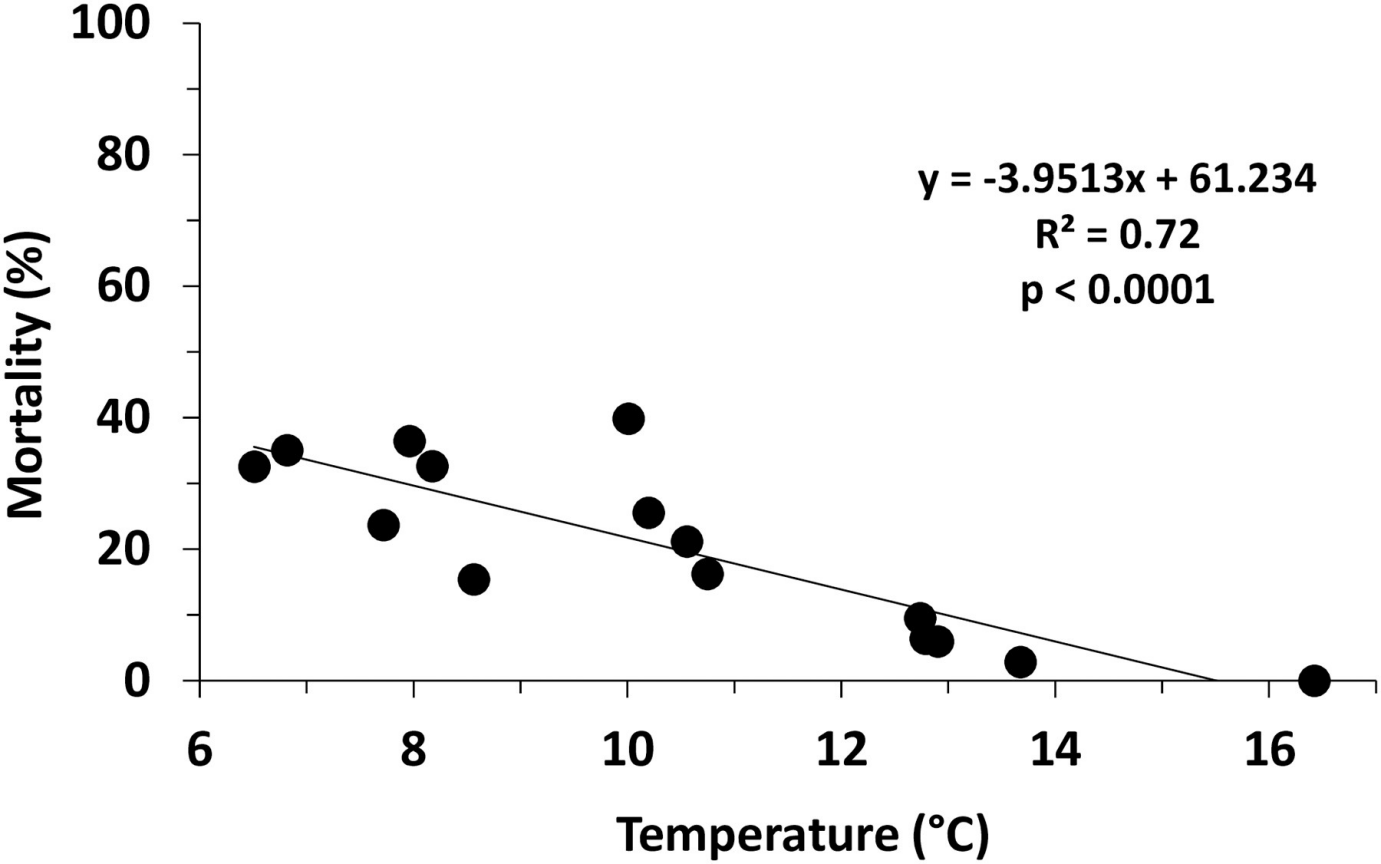
Temperature-dependent mortality of *Xylosandrus compactus* overwintering adults on carob twigs in Sicily (Southern Italy) considering the proportion of dead adults as a function of the minimum temperature trend.

The highest percentage of *X*. *compactus* dead adults, inside the infested twigs, was recorded from December to March (Fig 3), when the minimum temperatures (weekly mean) were consistently lower than 10 °C. The mortality peak (39.82% dead adults) occurred in the second half of January after several weeks with minimum daily temperatures lower than 5 °C. Results of the linear regression model (Fig 4) of the proportion of dead adults as a function of the minimum temperature trend showed that the temperature affected significantly the mortality of overwintering adults (R^2^ = 0.72; *F*_*1*,*13*_ = 33.434; *p* < 0.001).

### Fungal isolation, identification and characterization

A total of 54 fungal isolates belonging to different genus and species were obtained from galleries and beetles, and were identified using morphological and/or molecular analyses. In total, eight different species were associated with the beetle and/or galleries. Based on culture morphology, most isolates (13) were identified as *A*. *xylebori* and produced aleurioconidiophore with single aleurioconidium, in agreement with the description of this species by Brader (1964) and von Arx & Hennebert (1965) [52, 53].

The following species were identified from the other isolates: *F*. *solani* (9 isolates), *Xenoacremonium recifei* (10), *Clonostachys rosea* (12), *Acremonium* sp. (1), *Cytospora* sp. (4), *Aureobasidium pullulans* (3), and *Penicillium* sp. (2). To confirm the taxonomic identification, BLAST searches were performed for representative isolates of each species. ITS sequences of isolates CR 32 and CR 18 showed 99.6% and 100% homology with the holotype strain of *A*. *xylebori* (CBS 110.61) and *F*. *solani* (FKKM2), respectively. Similarly, the ITS sequences of CR 31 and CR 58 showed 99.2% and 100% identity with *X*. *recifei* (CBS 137.35) and *C*. *rosea* (CBS 149.52), respectively.

*Fusarium* and *Ambrosiella* species were the most prevalent isolates from galleries, i.e., 25.71% and 37.14%, respectively. On the other hand, *X*. *recifei* and *C*. *rosea* were isolated from both galleries and beetles. Occasionally, *Acremonium* sp., *Cytospora* sp. and *Penicillium* sp. were obtained from galleries, while *A*. *pullulans* were obtained from beetles.

## Discussion

This study provides the first data on the population structure and dynamics of *X*. *compactus* in Europe, and specifically on carob trees growing in Sicily (Southern Italy). The results of the annual beetle population trend show that, in the monitored environment, *X*. *compactus* overwinters inside twigs as adult and brood production begins in spring, after female emergence. Different generations were thus identified thanks to the study of the proportion of biological stages over the seasons (Fig 1). Egg peaks occurred in early May, mid-June, late July, early September and early October. These findings strongly suggest that the pest completed five generations, being active and reproducing from April and starting to overwinter in late autumn. The same number of peaks was found for the larval stage, and the time interval between adult peaks (about 6 weeks) was sufficient for *X*. *compactus* to complete its biological cycle, to find a new host plant/twig and establish a new gallery. In agreement with our findings, Ngoan et al. (1976) and Hara (1977), found that the *X*. *compactus* life cycle, from egg to adult, lasted 28.5 days [38, 54]. In addition, the female progeny left parental galleries from 7 to 9 days after pupal ecdysis, starting to lay eggs from 4 to 14 days after initial boring in a new twig [39].

The results of this study support the data obtained by sampling flying *X*. *compactus* adults using ethanol-baited traps in an earlier study by Gugliuzzo et al. (2019) [29]. These authors reported that the first flight of the year occurred in April, with temperatures consistently higher than 20 °C, and that the flight activity stopped in autumn, when the daily mean temperature decreased rapidly. In addition, the presence of only adults inside the twigs during the cold season confirms that the pest overwintered as adults, in line with a study that investigated the unusual behaviour of beetles on carob branches and trunks [30]. Sheltered and inactive groups of *X*. *compactus* adults were also found by Pennacchio et al. (2012) after a survey conducted on infested laurel plants in north western Italy [27]. Similarly, in Florida *X*. *compactus* were found to overwinter as adults inside successfully-infested twigs of flowering dogwood [38]. On the other hand, in Uganda the pest infesting robust a coffee was continuously active and all the biological stages occurred throughout the year [55].

Several studies report that this species prefers to attack small twigs and lateral small branches with a diameter <7 mm [22, 38, 56]. Our results showed that the mean infested diameter of carob twigs varied significantly over the seasons, reaching the minimum and maximum values during the summer (<4 mm) and the winter (>10 mm), respectively. In addition, during the cold season, the percentage of dead adults correlated negatively with the minimum temperature trend. This suggests that thicker twigs may represent a good shelter for *X*. *compactus* in order to survive the coldest periods. However, specific investigations are needed to verify these hypotheses. The beetle’s movement to larger twigs before overwintering indicates that pest control should be carried out by pruning and disposing the pruned material. Likewise, the preference shown by the beetle for small twigs during the summer suggests these twigs should be monitored and should be the focus of any possible control strategies.

In our study, the number of adults found inside carob galleries with only *X*. *compactus* adults ranged from 6 to 28 individuals per gallery. Considering galleries infested by both adults and young instars, other authors have reported that the number of specimens/gallery ranged from 3 to 36 on *L*. *nobilis* [24], 1 to 40 on *Cornus florida* L. [38] and 1 to 41 on *Coffea canephora* P. [22]. Similar data were also found by Gugliuzzo et al. (2019) on infested large branches and trunks of carob [29], where the mean number of adults/gallery was 19.98.

We found that the symbiotic fungus *A*. *xylebori* was the main ambrosia species associated with the black twig borer and isolated from galleries, as reported by Vannini et al. (2017) in Italy [26]. This species has been isolated from several host species in association with *X*. *compactus* worldwide [46, 57–60] and it has also been described in association with other beetle species, including congeneric *taxa* [61] and other *taxa* that are more distantly related [62]. Von Arx & Hennebert (1965) designated a type for the genus and species based on Brader’s isolate (CBS 110.61) [53]. In addition, as reported by Mayers et al. [60], Brader [52] and von Arx & Hennebert [53] described two types of aleurioconidiophores produced by the *Ambrosiella* species: one with disarticulating monilioid conidiophore cells, breaking off with attached aleurioconidia, and a second, straight, hyphoid aleurioconidiophore with a single, attached aleurioconidium. *Ambrosiella xylebori* has been described with the second conidiophore type.

Similarly, *A*. *xylebori* isolates in our collection produced single aleurioconidia from simple aleurioconidiophores, which likely do not disarticulate, and ITS sequences showed a high homology with the holotype strain (CBS 110.61). Although the role of these fungi still needs to be determined, some knowledge is already available in literature. *Ambrosiella xylebori* is a primary symbiotic fungus which is typically transported in the mycetangium of *X*. *compactus* and supports insect growth in the host tree. All known *Ambrosiella* spp. produce a fruity aroma [63], and these chemical volatiles may play a key role in attracting ambrosia beetles within the galleries [64].

In addition to the primary symbiont, some often non-mycangial fungi, are also associated with ambrosia beetles [46]. In this study, we isolated a *Fusarium* species from infested galleries, here identified as *F*. *solani*. *Fusarium solani* is a name given to a complex “*Fusarium solani* Species Complex” (FSSC) of over 45 morphologically cryptic species [65] and a recent study showed that FSSC is actually another genus of the Nectriaceae family named *Neocosmospora* [66]. Members of this genus have been reported in association with *X*. *compactus* and other ambrosia beetles, and they are often reported as pathogenic to the host tree [22, 38, 44, 67], and to other woody crops (i.e., avocado) in Sicily [68]. A recent study also demonstrated that *X*. *compactus* females are attracted to several bioactive volatile compounds released by *F*. *solani* [67]. Wood tunneling by *X*. *compactus* females interrupts the transmission of water and nutrients within the plant, leading to wilting of the infested plant part within weeks [22, 38]. In addition, secondary pathogens, fungal symbionts, and a plant response likely contribute to the dieback [69].

The other fungi isolated from the sampled galleries were *Acremonium* sp., already reported by Bateman et al. [46], and *Penicillium* sp., reported in association with *X*. *germanus* [19]. The latter appears to be passively introduced during gallery excavation [70]. By contrast, to the best of our knowledge, this study reports for the first time the association of *Cytospora* sp., *A*. *pullulans*, *X*. *recifei* and *C*. *rosea* with this ambrosia beetle. *Cytospora* species are canker and dieback pathogens of woody hosts [71, 72], whereas *A*. *pullulans*, *X*. *recifei* and *C*. *rosea* have not been reported as pathogens, and their role still needs to be determined and merits further study. As reported by Hofstetter et al. (2006), some fungi consistently carried by bark beetles have been found to be commensal or even antagonistic [73]. In addition, the low isolation frequency of some isolates suggests either contamination or incidental association related to abiotic factors, which may influence the presence of fungi associated with *X*. *compactus* and the occurrence during the isolation process [42, 43, 59]. Considering that the fungi identification of this study is based on ITS sequences, a companion investigation is currently ongoing with the aim of sequencing additional loci of the fungal isolates associated with *X*. *compactus*. Such further study will focus on molecular characterization and multi-locus phylogeny allowing a definitive fungi identification at the species level.

Taken as a whole, our findings highlight that in this newly colonized environment the pest is able to complete five generations per year. Moreover, our results allowed to intercept the key steps of the population phenology and the spring first flight of the beetle. Thus, these data provide useful knowledge for ameliorating the monitoring and sustainable control of *X*. *compactus*, namely, winter pruning and removal of infested twigs coupled with summer monitoring of younger twigs may represent an effective and environmentally-friendly strategy for *X*. *compactus* control on carob trees.

This study was carried out in one of the very first areas (south east Sicily, Southern Italy) among those recently invaded by *X*. *compactus* in Europe, and on the main host plant severely affected by this pest in the described area, i.e. carob tree. However, considering the high invasive potential and wide host range of *X*. *compactus*, further investigations on its population dynamics and symbiotic associations in other environments and host plants would provide more definitive insights into the invasion process, multitrophic interactions and real potential damage of *X*. *compactus* in the Mediterranean Basin.

## Supporting information

S1 Table Raw datasets presented without restriction. (XLSX)
